# Current News

**Published:** 1904-08

**Authors:** 


### Current News.

### FOURTH INTERNATIONAL DENTAL CONGRESS, ST. LOUIS, MO., AUGUST 29 TO SEPTEMBER 3, 1904.

*Committee of Organization.*—H. J. Burkhart, Chairman, Batavia, N. Y.; E. C. Kirk, Secretary, Lock Box 1615, Philadelphia, Pa.; R. H. Hofheinz, Wm. Carr, W. E. Boardman, V. E. Turner, J. Y. Crawford, M. F. Finley, J. W. David, Wm. Crenshaw, Don M. Gallie, G. V. I. Brown, A. H. Peck, J. D. Patterson, B. L. Thorpe.

The Department of Congresses of the Universal Exposition, St. Louis, 1904, has nominated the Committee of Organization of the Fourth International Dental Congress which was appointed by the National Dental Association, and has instructed the committee thus appointed to proceed with the work of organization of said Congress.

Pursuant to the instructions of the Director of Congresses of the Universal Exposition, 1904, the Committee of Organization presents the subjoined outline of the plan of organization of the Dental Congress.

The Congress will be divided into two departments: Department A—SCIENCE (divided into four sections). Department B—APPLIED SCIENCE (divided into six sections).

### DEPARTMENT A—SCIENCE.

I. Anatomy, Physiology, Histology, and Microscopy. Chairman, M. H. Cryer, 1420 Chestnut Street, Philadelphia, Pa.

II. Etiology, Pathology, and Bacteriology. Chairman, R. H. Hofheinz, Chamber of Commerce, Rochester, N. Y.

III. Chemistry and Metallurgy. Chairman, J. D. Hodgen, 1005 Sutter Street, San Francisco, Cal.

IV. Oral Hygiene, Prophylaxis, Materia Medica and Therapeutics, and Electro-therapeutics. Chairman, A. H. Peck, 92 State Street, Chicago, Ill.

### DEPARTMENT B—APPLIED SCIENCE.

V. Oral Surgery. Chairman, G. V. I. Brown, 445 Milwaukee Avenue, Milwaukee, Wis.

VI. Orthodontia. Chairman, E. H. Angle, 1023 North Grand Avenue, St. Louis, Mo.

VII. Operative Dentistry. Chairman, C. N. Johnson, Marshall Field Building, Chicago, Ill.

VIII. Prosthesis. Chairman, C. R. Turner, Thirty-third and Locust Streets, Philadelphia, Pa.

IX. Education, Nomenclature, Literature, and History. Chairman, Truman W. Brophy, Marshall Field Building, Chicago, Ill.

X. Legislation. Chairman, Wm. Carr, 35 West Forty-sixth Street, New York, N. Y.

COMMITTEES.

Following are the committees appointed:

*Finance.*—Chairman, C. S. Butler, 680 Main Street, Buffalo, N. Y.

*Programme.*—Chairman, A. H. Peck, 92 State Street, Chicago, Ill.

*Exhibits.*—Chairman, D. M. Gallie, 100 State Street, Chicago, Ill.

*Transportation.*—(To be appointed.)

*Reception.*—Chairman, B. Holly Smith, 1007 Madison Avenue, Baltimore, Md.

*Registration.*—Chairman, B. L. Thorpe, 3666 Olive Street, St. Louis, Mo.

*Printing and Publication.*—Chairman, W. E. Boardman, 184 Boylston Street, Boston, Mass.

*Conference with State and Local Dental Societies.*—Chairman, J. A. Libbey, 524 Penn Avenue, Pittsburg, Pa.

*Dental Legislation.*—Chairman, Wm. Carr, 35 West Forty-sixth Street, New York, New York.

*Auditing.*—(Committee of Organization.)

*Invitation.*—Chairman, L. G. Noel, 527½ Church Street, Nashville, Tenn.

*Membership.*—Chairman, J. D. Patterson, Keith and Perry Building, Kansas City, Mo.

*Educational Methods.*—Chairman, T. W. Brophy, Marshall Field Building, Chicago, Ill.

*Oral Surgery.*—Chairman, G. V. I. Brown, 445 Milwaukee Avenue, Milwaukee, Wis.

*Prosthetic Dentistry.*—Chairman, C. R. Turner, Thirty-third and Locust Streets, Philadelphia, Pa.

*Local Committee of Arrangements and Reception.*—Chairman, Wm. Conrad, 3666 Olive Street, St. Louis, Mo.

*Essays.*—Chairman, Wilbur F. Litch, 1500 Locust Street, Philadelphia, Pa.

*History of Dentistry.*—Chairman, Wm. H. Trueman, 900 Spruce Street, Philadelphia, Pa.

*Nomenclature.*—Chairman, A. H. Thompson, 720 Kansas Avenue, Topeka, Kan.

*Promotion of Appointment of Dental Surgeons in the Armies and Navies of the World.*—Chairman, Williams Donnally, 1018 Fourteenth Street N. W., Washington, D. C.

*Care of the Teeth of the Poor.*—Chairman, Thomas Fillebrown, 175 Newbury Street, Boston, Mass.

*Etiology, Pathology, and Bacteriology.*—Chairman, R. H. Hofheinz, Chamber of Commerce, Rochester, N. Y.

*Prize Essays.*—Chairman, James Truman, 4505 Chester Avenue, Philadelphia, Pa.

*Oral Hygiene, Prophylaxis, Materia Medica and Therapeutics, and Electro-therapeutics.*—Chairman, A. H. Peck, 92 State Street, Chicago, Ill.

*Operative Dentistry.*—Chairman, C. N. Johnson, Marshall Field Building, Chicago, Ill.

*Resolutions.*—Chairman, J. Y. Crawford, Jackson Building, Nashville, Tenn.

*Clinics.*—Chairman, J. P. Gray, 212 North Spruce Street, Nashville, Tenn.

*Nominations.*—Chairman, A. H. Peck, 92 State Street, Chicago, Ill.; W. E. Boardman, 184 Boylston Street, Boston, Mass.; M. R. Windhorst, 3518 Morgan Street, St. Louis, Mo.; Wm. Conrad, 3666 Olive Street, St. Louis, Mo.

*Ad Interim.*—Chairman, G. V. I. Brown, 445 Milwaukee Avenue, Milwaukee, Wis.

The officers of the Congress, president, vice-presidents, secretary, and treasurer, will be elected by the Congress at large at the time of the meeting, and will be nominated by the Nominating Committee.

The Fourth International Dental Congress, which will be held August 29 to September 3, inclusive, 1904, will be representative of the existing status of dentistry throughout the world. It is intended further that the Congress shall set forth the history and material progress of dentistry from its crude beginnings through its developmental stages, up to its present condition as a scientific profession.

The International Dental Congress is but one of the large number of congresses to be held during the period of the Louisiana Purchase Exposition, and these in their entirety are intended to exhibit the intellectual progress of the world, as the Exposition will set forth the material progress which has taken place since the Columbian Exposition in 1893.

It is important that each member of the dental profession in America regard this effort to hold an International Dental Congress as a matter in which he has an individual interest, and one which he is under obligation to personally help towards a successful issue. The dental profession of America has not only its own professional record to maintain with a just pride, but, as it is called upon to act the part of host in a gathering of our colleagues from all parts of the world, it has to sustain the reputation of American hospitality as well.

The Committee of Organization appeals earnestly to each member of the profession to do his part in making the Congress a success. Later bulletins will be issued setting forth the personnel of the organization and other particulars, when the details have been more fully arranged.

H. J. Burkhart, *Chairman.*
E. C. Kirk, *Secretary.*

Approved:
Howard J. Rogers,
*Director of Congresses.*
David R. Francis,
*President of Exposition.*

COMMITTEE ON STATE AND LOCAL ORGANIZATIONS.

(J. A. Libbey, Chairman, 524 Penn Avenue, Pittsburg, Pa.)

The Committee on State and Local Organizations is a committee appointed by the Committee of Organization of the Fourth International Congress with the object of promoting the interests of the Congress in the several States of the Union. Each member of the committee is charged with the duty of receiving applications for membership in the Congress under the rules governing membership as prescribed by the Committee on Membership and approved by the Committee of Organization. These rules provide that *membership in the Congress shall be open to all reputable legally qualified practitioners of dentistry.* Membership in a State or local society is not a necessary qualification for membership in the Congress.

Each State chairman, as named below, is furnished with official application blanks and is authorized to accept the membership fee of ten dollars from all eligible applicants within his State. The State chairman will at once forward the fee and official application with his indorsement to the chairman of the Finance Committee, who will issue the official certificate conferring membership in the Congress. No application from any of the States will be accepted by the chairman of the Finance Committee unless approved by the State chairman, whose indorsement is a certification of eligibility under the membership rules.

A certificate of membership in the Congress will entitle the holder thereof to all the rights and privileges of the Congress, the right of debate, and of voting on all questions which the Congress will be called upon to decide. It will also entitle the member to one copy of the official transactions when published and to participation in all the events for social entertainment which will be officially provided at the time of the Congress.

The attention of all reputable legally qualified practitioners of dentistry is called to the foregoing plan authorized by the Committee of Organization for securing membership in the Congress, and the committee earnestly appeals to each eligible practitioner in the United States who is interested in the success of this great international meeting to make application at once through his State chairman for a membership certificate. By acting promptly in this matter the purpose of the committee to make the Fourth International Dental Congress the largest and most successful meeting of dentists ever held will be realized, and the Congress will thus be placed upon a sound financial basis.

Let every one make it his individual business to help at least to the extent of enrolling himself as a member and the success of the undertaking will be quickly assured. Apply at once to your State chairman. The State chairmen already appointed are:

*General Chairman.*—J. A. Libbey, 524 Penn Avenue, Pittsburg, Pa.

*Alabama.*—H. Clay Hassell, Tuscaloosa.

*Arkansas.*—W. H. Buckley, 510½ Main Street, Little Rock.

*California.*—J. L. Pease, 1016 Clay Street, Oakland.

*Colorado.*—H. A. Fynn, 500 California Building, Denver.

*Connecticut.*—Henry McManus, 80 Pratt Street, Hartford.

*Delaware.*—C. R. Jeffries, New Century Building, Wilmington.

*District of Columbia.*—W. N. Cogan, The Sherman, Washington.

*Florida.*—W. G. Mason, Tampa.

*Georgia.*—H. H. Johnson, Macon.

*Hawaii.*—M. E. Grossman, Box 744, Honolulu.

*Idaho.*—J. B. Burns, Payette.

*Illinois.*—J. E. Hinkins, 131 East Fifty-third Street, Chicago.

*Indiana.*—H. C. Kahlo, 115 East New York Street, Indianapolis.

*Iowa.*—W. R. Clark, Clear Lake.

*Kansas.*—G. A. Esterly, Lawrence.

*Kentucky.*—H. B. Tileston, 314 Equitable Building, Louisville.

*Louisiana.*—Jules J. Sarrazin, 108 Bourbon Street, New Orleans.

*Maine.*—H. A. Kelley, 609 Congress Street, Portland.

*Maryland.*—W. G. Foster, 813 Eutaw Street, Baltimore.

*Massachusetts.*—M. C. Smith, 3 Lee Hall, Lynn.

*Michigan.*—G. S. Shattuck, 539 Fourth Avenue, Detroit.

*Minnesota.*—C. A. Van Duzee, 51 Germania Bank Building, St. Paul.

*Mississippi.*—W. R. Wright, Jackson.

*Missouri.*—J. W. Hull, Altman Building, Kansas City.

*Montana.*—G. E. Longeway, Great Falls.

*Nebraska.*—H. A. Shannon, 1136 "O" Street, Lincoln.

*New Hampshire.*—E. C. Blaisdell, Portsmouth.

*New Jersey.*—Alphonso Irwin, 425 Cooper Street, Camden.

*New York.*—B. C. Nash, 142 West Seventy-eighth Street, New York City.

*North Carolina.*—C. L. Alexander, Charlotte.

*Ohio.*—Henry Barnes, 1415 New England Building, Cleveland.

*Oklahoma.*—T. P. Bringhurst, Shawnee.

*Oregon.*—S. J. Barber, Macleay Building, Portland.

*Pennsylvania.*—H. E. Roberts, 1516 Locust Street, Philadelphia.

*Rhode Island.*—D. F. Keefe, 315 Butler Exchange, Providence.

*South Carolina.*—J. T. Calvert, Spartanburg.
*South Dakota.*—E. S. O'Neil, Canton.
*Tennessee.*—W. P. Sims, Jackson Building, Nashville.
*Texas.*—J. G. Fife, Dallas.
*Utah.*—W. L. Ellerbeck, 21 Hooper Building Salt Lake City.
*Vermont.*—S. D. Hodge, Burlington.
*Virginia.*—F. W. Stiff, 2101 Churchill Avenue, Richmond.
*Washington.*—G. W. Stryker, Everett.
*West Virginia.*—H. H. Harrison, 1141 Main Street, Wheeling.
*Wisconsin.*—A. D. Gropper, 401 East Water Street, Milwaukee.

For the Committee of Organization,
EDWARD C. KIRK,
*Secretary.*

---

### PROGRAMME OF THE FOURTH INTERNATIONAL DENTAL CONGRESS.

### DEPARTMENT A—SCIENCE.

*Section I.—Anatomy, Physiology, Histology, and Microscopy.* (*Chairman, Dr. M. H. Cryer, 1420 Chestnut Street, Philadelphia, Pa.*)

Florestan Aguilar, Madrid, Spain, "General Anæsthesia by Somnoform."

G. G. Campion, Manchester, England, "Determining the Actual Path and Extent of Movement of the Mandible Condyle in the Living Subject."

D. E. N. Caush, Brighton, England, "Is there Uncalcified Tissue in the Enamel?"

M. H. Cryer. (Subject to be announced.)

W. T. Eckley, "Phylogenetic Evidence regarding the Function of the Accessory Sinuses in Man."

John E. Grevers, Amsterdam, Holland, "Anatomy of the Facial Skull—Normal and in Mouth-Breathers."

————, "Geometrical Construction of the Mandible."

————, "Behavior of the Teeth under Polarized Light."

A. Hopewell-Smith, London, England. (Subject to be announced.)

Eugene S. Talbot, Chicago, Ill. (Subject to be announced.)

A. H. Thompson, Topeka, Kan., "Ethnographic Odontography: The Mound-Builders and the Pre-Indian Peoples of the Mississippi Valley."

J. G. Turner, London, England. (Subject to be announced.)

Arthur S. Underwood, London, England. (Subject to be announced.)

O. Walkhoff, Munich, Germany, "Concerning the Crania of Diluvial Peoples." (Illustrated with lantern slides.)

*Section II.—Etiology, Pathology, and Bacteriology. (Chairman, Dr. R. H. Hofheinz, Chamber of Commerce, Rochester, N. Y.)*

C. F. W. Bödecker, Berlin, Germany, "Percussion in Dental Diagnosis."

G. W. Cook, Chicago, Ill., "The Effects of Chemical Agents on Bacteria with Relation to the Saliva."

Samuel A. Hopkins, Boston, Mass., "Application of the Results of Research Work to Daily Practice."

W. H. G. Logan, Chicago, Ill., "A Consideration of Some of the Etiological Factors that produce Tissue Changes of the Alveolar Process and Overlying Soft Parts."

Jos. P. Michaels, Paris, France, "Sialology: Differential Analyses; Elements of Value in Medical Diagnosis."

W. D. Miller, Berlin, Germany, "Researches relating to Various Pathological Processes in the Teeth."

Louis Ottofy, Manila, P. I., "Observations on the Causes of Erosion: (*a*) Erosio Areca (betel erosion); (*b*) Erosio Orientalis."

J. D. Patterson, Kansas City, Mo. (Subject to be announced.)

M. L. Rhein, New York, N. Y. (Subject to be announced.)

Oskar Römer, Strasburg, Germany, "Some Patho-histological Observations on Pyorrhœa Alveolaris."

D. D. Smith, Philadelphia, Pa., "Pericemental Abscess."

Eugene S. Talbot, Chicago, Ill., "Constitutional Causes of Tooth-Decay."

F. Vicentini, Chieti, Italy, "Leptothrix Racemosa."

*Section III.—Chemistry and Metallurgy. (Chairman, Dr. J. D. Hodgen, 1005 Sutter Street, San Francisco, Cal.)*

J. P. Buckley, Chicago, Ill., "Chemistry of Pulp-Decomposition."

H. C. Carel, Minneapolis, Minn. (Subject to be announced.)

J. D. Hodgen, San Francisco, Cal., "Chemistry and Dentistry."

Hof-Zahnarzt W. Pfaff, Dresden, Germany, "Das Aluminium und seine Anwendbarkeit in Allgemeinen."

R. W. Simon, Boston, Mass. (Subject to be announced.)

Herbert L. Wheeler, "The Chemistry of Porcelain."

*Section IV.—Oral Hygiene, Prophylaxis, Materia Medica and Therapeutics, and Electro-Therapeutics. (Chairman, Dr. A. H. Peck, 92 State Street, Chicago, Ill.)*

Samuel Taylor Bassett, St. Louis, Mo., "Application of Electro-Therapeutics to Dental Surgery."

L. P. Bethel, Columbus, Ohio, "Some Results from Dental and Oral Prophylaxis."

Julio Endelman, Philadelphia, Pa., "Contribution to the Therapeutics of Post-Extraction Accidents."

Richard Grady, Annapolis, Md., "Oral Hygiene: Mastication."

J. E. Hinkins, Chicago, Ill. (Subject to be announced.)

Edward Hoffmeister, Baltimore, Md., "Materia Medica."

Prof. Dr. Jessen, Strasburg; Dr. Loos, Vienna, and Zahnarzt Georg Schlaeger: I. "Zahn-hygiene in Schule and Heer." II. Eine Wandtafel für den Ausschauungsunterricht in der Schule in Farben, "Gesunde und Kranke Zähne." III. Eine Wandtafel, ii. Auflage auch farbig, "Die Zähne und ihre Pfläge."

Weston A. Price, Cleveland, Ohio. (Subject to be announced.)

E. Sauvez, Paris, France, "Study of the Various Means of inducing Local Anæsthesia for Extraction of the Teeth."

Zahnarzt Dr. Schaeffer-Stuckert, Frankfort-on-Main, Germany, "Paranephrin Bitsert: A New Preparation of Kidney Atrabilarian in Connection with Local Anæsthetics in Dentistry."

Edward Schlinkmann, Baltimore, Md., "Electric Absorption in Therapy."

C. R. Taylor, Streator, Ill. (Subject to be announced.)

### DEPARTMENT B—APPLIED SCIENCE.

*Section V.—Oral Surgery. (Chairman, Dr. G. V. I. Brown, 445 Milwaukee Street, Milwaukee, Wis.)*

Chairman's address, G. V. I. Brown, Milwaukee, Wis., "Oral Surgery: Its Relations to General Surgery and Dentistry."

T. W. Brophy, Chicago, Ill., "Necessity for Early Operation for Cleft Palate."

T. L. Gilmer, Chicago, Ill., "The Teaching of Oral Surgery in Our Dental Schools."

H. H. Grant, Louisville, Ky., "Solid Tumors involving the Body or Ramus of the Inferior Maxillary Bone."

J. G. Kiernan, Chicago, Ill., "Embryogenetic, Congenital, and Acquired Stomato-Neurologic Relations."

A. H. Levings, Milwaukee, Wis., "Importance and Methods of Early Diagnosis of Malignant Growths affecting the Maxillary Bone."

J. S. Marshall, San Francisco, Cal., "Fractures of the Mandible and their Treatment."

E. S. Talbot, Chicago, Ill., "Etiology of Cleft Palate and Harelip."

*Section VI.—Orthodontia.* (*Chairman, Dr. Edward H. Angle, 1023 North Grand Avenue, St. Louis, Mo.*)

Edward H. Angle, St. Louis, Mo., "Malocclusion: Class II. and its Divisions."

G. V. I. Brown, Milwaukee, Wis. (Subject to be announced.)

L. C. Bryan, Basel, Switzerland, "Nature as a Regulator, and our Duty as her Assistants."

Calvin S. Case, Chicago, Ill., "Principles and Methods of Retention in Orthodontia."

M. Chiwaki, Tokyo, Japan. (Subject to be announced.)

Wm. Slocum Davenport, Paris, France, "Contribution to the Treatment of Short Bite and Jump Bite Cases."

Robert Dunn, San Francisco, Cal., "Mesial Position of the First Molars in Class I."

John E. Grevers, Amsterdam, Holland, "Proposal for an International Nomenclature for the Various Forms of Malocclusion."

Chas. A. Hawley, Columbus, Ohio, "Method of determining the Normal Arch, and its Application in Orthodontia."

Francisque Martin, Lyons, France, "The Correction of Deformities in Fractures of the Nose."

R. Ottolengui, New York, N. Y., "Spreading the Maxillæ *versus* Spreading the Arch."

W. Booth Pearsall, Dublin, Ireland, "Irish Types of Malocclusion."

Herbert A. Pullen, Buffalo, N. Y. (Subject to be announced.)

Jose J. Rojo, Mexico City, Mexico, "Study of the Etiology of Anomalies in Human Teeth."

Dr. Schroeder, Greifswald, Germany, "Prognathous Forms and their Orthopædic Treatment."

A. Hopewell-Smith, London, England. (Subject to be announced.)

J. Sim Wallace, London, England, "Nasal Obstructions and Mouth-Breathing, with Special Reference to Malocclusion of the Teeth."

S. Merrill Weeks, Philadelphia, Pa. (Subject to be announced.)

Edmund Wuerpel, St. Louis, "Art."

Franz Zeliska, Vienna, Austria. (Subject to be announced.)

*Section VII.—Operative Dentistry. (Chairman, Dr. C. N. Johnson, Marshall Field Building, Chicago, Ill.)*

E. A. Bogue, New York, N. Y. (Subject to be announced.)

Jas. M. Magee, St. Johns, N. B., "The Instrumentation and Filling of Crooked Root-Canals." (Illustrated.)

Sylvester Moyer, Galt, Ont., "The Enamel and its Consideration in Cavity Preparation."

C. G. Myers, Cleveland, Ohio. (Subject to be announced.)

Garrett Newkirk, Los Angeles, Cal., "The Whole Question of Matrices and their Uses."

Frank L. Platt, San Francisco, Cal. (Subject to be announced.)

Geo. C. Poundstone, Chicago, Ill., "The Cement Problem in Inlay Work."

M. L. Rhein, New York, N. Y. (Subject to be announced.)

Arthur Scheuer, Teplitz, Austria, "Tin-Cement, Sponge Tin: Two New Filling-Materials and their Uses."

E. K. Wedelstaedt, St. Paul, Minn., "Gold-and-Tin."

H. L. Wheeler, New York, N. Y. (Subject to be announced.)

*Section VIII.—Prosthesis. (Chairman, Dr. C. R. Turner, Thirty-third and Locust Streets, Philadelphia, Pa.)*

L. W. Baker, Boston, Mass. (Subject to be announced.)

George Brunton, Leeds, England. (Subject to be announced.)

Reuben C. Brophy, Chicago, Ill., "Rationale of the Use of Materials for Base-Plates in the Construction of Artificial Dentures."

Calvin S. Case, Chicago, Ill., "The Mechanical Treatment of Cleft Palate."

Edw. G. Christensen, Drommen, Norway, "Which is the Ideal Crown,—the Banded Crown or the Crown without Band?"

B. J. Cigrand, Chicago, Ill., "Facial Guide Lines as taught by Artists."

Bernard Frank, Amsterdam, Holland, "A New Articulator on Anatomical Principles."

Hart J. Goslee, Chicago, Ill., "Porcelain Crowns."

F. H. Mamlock, Berlin, Germany, "(*a*) Ueber Porzellanstiftzähne. (*b*) Ueber Magnalium Prothesen und ihre Herstellung nach Dr. Eug. Müllerschen Gummidrucksystem."

Francisque Martin, Lyons, France, "Immediate Prosthesis after the Method of Dr. Claude Martin."

Joseph Nolin, Montreal, Canada, "The Decline of Æstheticism in Prosthesis."

B. Platschick, Paris, France, "(*a*) Influence de Fauchard sur la Prothèse dentaire. (b) Les dents à tube en général et leur emploi special pour les pièces à gencive continue. (*c*) Contribution à l'étude des Couronnes."

Jas. H. Prothero, Chicago, Ill. (Subject to be announced.)

Rudolph Weiser, Vienna, Austria, "Some Cases illustrating the Possibilities of Prosthetic Dentistry."

E. Lloyd Williams, London, England. (Subject to be announced.)

Geo. H. Wilson, Cleveland, Ohio. (Subject to be announced.)

*Section IX.—Education, Nomenclature, Literature, and History. (Chairman, Dr. Truman W. Brophy, Marshall Field Building, Chicago, Ill.)*

M. Chiwaki, Tokyo, Japan, "Dentistry in Japan."

Charles Godon, Paris, France, "Educational Standards of Europe."

S. H. Guilford, Philadelphia, Pa., "Nomenclature."

A. W. Harlan, New York, N. Y., "Dental Literature."

A. O. Hunt, Omaha, Neb., "The Count System of Students' Credits."

Chas. McManus, Hartford, Conn., "International Character of the Early Development of Dentistry in America."

Louis Ottofy, Manila, P. I. (Subject to be announced.)

Jose J. Rojo, City of Mexico, Mexico, "Historical Annotations and Present Condition of Dental Education in the City of Mexico."

B. L. Thorpe, St. Louis, Mo., "History of American Dentistry."

James Truman, Philadelphia, Pa., "Dental Education from the View of Experience."

*Section X.—Legislation. (Chairman, Dr. Wm. Carr, 35 West Forty-sixth Street, New York, N. Y.)*

(Not received.)

CLINICS.

(Reports for the East and South have not yet been received.)

*Porcelain.*—C. C. Allen, Kansas City, Md.; W. V.-B. Ames, Chicago, Ill.; E. H. Ball, Tama, Iowa; F. E. Cheeseman, Chicago, Ill.; W. A. Coston, Fort Scott, Kan.; W. H. Cudworth, Milwaukee, Wis.; A. W. Dana, Burlington, Iowa; S. F. Duncan, Joliet, Ill.; Adam Flickinger, St. Louis, Mo.; V. H. Frederick, St. Louis, Mo.; H. J. Goslee, Chicago, Ill.; F. B. James, Wilton Junction, Iowa; Robt. LeCron, St. Louis, Mo.; L. A. Meyer, Oconomowoc, Wis.; J. E. Nyman, Chicago, Ill.; W. T. Reeves, Chicago, Ill.; W. H. Taggart, Chicago, Ill.; C. N. Thompson, Chicago, Ill.; J. E. Wait, Superior, Neb.; C. M. Work, Ottumwa, Iowa.

*Gold Inlays.*—F. T. Breene, Iowa City, Iowa; O. H. Simpson, Dodge City, Kan.; C. N. Thompson, Chicago, Ill.; W. F. Whalen, Peoria, Ill.; C. H. Wright, Chicago, Ill.

*Surgery.*—T. W. Brophy, Chicago, Ill.; T. L. Gilmer, Chicago, Ill.; G. D. Moyer, Montevideo, Minn.

*Gold Fillings.*—A. G. Fee, Superior, Wis.; F. O. Hetrick, Ottowa, Canada; J. G. Pfaff, St. Louis, Mo.; C. H. Seeger, Manitowoc, Wis.; F. G. Van Stratum, Hurley, Wis.; J. W. Wick, St. Louis, Mo.; (G. V. Black Club) S. Bond, Anoka, Minn.; K. E. Carlson, St. Paul, Minn.; W. R. Clack, Clear Lake, Iowa; J. V. Conzett, Dubuque, Iowa; Wm. Finn, Cedar Rapids, Iowa; S. R. Holden, Duluth, Minn.; A. M. Lewis, Austin, Minn.; J. B. Pherrin, Central City, Iowa; G. A. Rawlings, Bismarck, N. D.; A. J. Schlueter, Aberdeen, S. D.; A. C. Searl, Owatonna, Minn.; J. F.

Wallace, Canton, Mo.; E. K. Wedelstaedt, St. Paul, Minn.; R. B. Wilson, St. Paul, Minn.

*Gold Crowns.*—J. G. Hollingsworth, Kansas City, Mo.; C. M. Work, Ottumwa, Iowa.

*Pyorrhœa Alveolaris.*—W. H. G. Logan, Chicago, Ill.; O. H. Manhard, St. Louis, Mo.; G. R. Richter, Milwaukee, Wis.; C. R. Taylor, Streator, Ill.

*Extracting.*—J. W. Slonaker, Chicago, Ill.

*Miscellaneous.*—H. F. Cassel, St. Louis, Mo., "Striking up partial gold plate with swaged enforcement single thickness gold."

W. L. Ellerbeck, Salt Lake City, Utah, "Electric furnace construction."

Otto J. Fruth, St. Louis, Mo., "Replantation with porcelain restoration."

R. R. Johnson, Great Falls, Neb., "Artificial velum and obturator."

Elgin MaWhinney, Chicago, Ill., "New drugs; valuable old ones; with therapeutics and indications for uses."

T .W. Pritchett, Whitehall, Ill., "Bonwill method of articulating full dentures."

J. B. Ridout, St. Paul, Minn., "New method of backing facings for Richmond crowns; new method of making continuous gum plate; method of putting gold corner or filling in porcelain tooth."

H. A. Shannon, Lincoln, Neb., "Æsthetic crowns and dummies."

C. O. Simpson, St. Louis, Mo., "Demonstrating strength and durability of amalgams and cements."

G. O. Sitherwood, Bloomington, Ill., "Soldering bands and fixtures; gold plating and adjusting."

W. R. Smith, Pawnee City, Neb., "Prosthetic."

Richard Summa, St. Louis, Mo., "Practical application of Angle fracture band."

S. H. Voyles, St. Louis, Mo., "Saddle bridge; pinless teeth."

E. R. Warner, Denver, Col., "Masticating force of human jaws, demonstrated by appliances."

*Subjects not given.*—C. S. Case, Chicago, Ill.; W. D. James, Tracy, Minn.; F. E. Roach, Chicago, Ill.; Jas. Weirick, St. Paul, Minn.

EXHIBITORS.

The following manufacturers and dealers have reserved space up to date:

The S. S. White Dental Manufacturing Company, Claudius Ash & Sons, Limited, H. D. Justi & Son, Kress & Owen Company ("Glyco-Thymoline"), J. W. Ivory (Specialties), Oakland Chemical Company, American Cabinet Company, Ransom & Randolph Company, Ammonol Company, John T. Nolde Dental Manufacturing Company, Hisey Manufacturing Company, E. De Trey & Sons, Johnson & Johnson, Detroit Dental Manufacturing Company, Harvard Company, Lee S. Smith & Son, Sanitol Chemical Laboratory Company, John T. Milliken Company, S. Eldred Gilbert Dental Manufacturing Company, Ritter Dental Manufacturing Company, Young Dental Manufacturing Company, McKesson & Robbins Chemical Company, Dentists' Supply Company, Pinches & Ely (Specialties), Frink & Young, W. V.-B. Ames, A. C. Clark & Co., Horlick's Food Company, Chas. H. Phillips Chemical Company, Whiteside Dental Manufacturing Company, Klewe & Company (Jenkins porcelain), R. C. Brophy, L. O. Green, L. D. Caulk, Dutro & Hewitt, Blair Dental Manufacturing Company, Peroxident Chemical Company, Goldsmith Bros., Adrian Rutherford.

NOTE.—The United States government will make an exhibit consisting of a complete dental outfit as furnished to the members of the Army Dental Corps.

---

### FOURTH INTERNATIONAL DENTAL CONGRESS BANQUET.

THE Fourth International Dental Congress Banquet will be held on September 1, 1904, at 8 P.M., in the Coliseum Building adjoining the Congress Hall. The price per plate is three dollars. It is requested that all who expect to attend will send their names and money to Dr. A. H. Fuller, P. O. Lock-Box 604, St. Louis, Mo., at once, and not later than August 20. Arrangements to pay can be made with Dr. A. H. Fuller at the time of registration, provided notice is given before August 20.

G. A. BOWMAN,
A. H. FULLER,
ADAM FLICKINGER,
*Banquet Committee.*

### UNIVERSAL EXPOSITION—HOTEL RATES.

### FOURTH INTERNATIONAL DENTAL CONGRESS.

(*Saint Louis, August 29 to September 3, 1904.*)

THE Local Committee and Bureau of Information wish to dispel from the minds of the profession and all persons that labor under any misconception that the rates of St. Louis Hotels are extortionate.

We have investigated the conditions and rates of the leading hotels of St. Louis, and notwithstanding the fact that this city is entertaining a World's Fair, the hotel rates are no higher than in other cities.

We will append a number of the leading hotels and rates of same, and call your attention that it is not required of you to put up at any of the hotels mentioned, as there are many hotels and boarding-houses in the city where rooms can be secured for from fifty cents to two dollars per day. The exact date should be stated in securing rooms.

We append a list of hotels and rates of same as follows:

*Southern Hotel.*—The American plan rate is $5 per day for room without bath and $6 a day for a room with bath. The rate is $10 per day if two persons occupy the room.

*Planters Hotel.*—Room without bath, occupied by one person, $3 to $4 a day. Same for two persons, $6 to $7 a day. Room with bath for one person, $4 to $5 a day, and for two persons $7 to $8 a day.

*Jefferson Hotel.*—Room without bath, for one person, $4 a day; when occupied by two persons, $6 a day. Room with bath for one person, $5 a day and up; when occupied by two or more persons, $7 a day and up.

*St. Nicholas Hotel.*—Room without bath, for one person, $2.50 to $3.50 a day; for two persons, $4 to $5 a day. Room with bath, for one person, $3 to $5 a day; for two persons, $5 to $7 a day.

*Lindell Hotel.*—Room without bath, for one person, $2 a day; for two persons, $3 a day. Room with bath, for one person, $3 a day; for two persons, $4 a day.

*Washington Hotel.*—Room without bath, for one, two, or three persons, $5 to $7 a day; room with bath, for one, two, or three persons, $8 a day.

*Laclede Hotel.*—Room without bath, for one person, $1.50 to $2 a day; for two persons, $3 a day; room with bath, for one person, $3 a day; for two persons, $5 a day.

*Terminal Hotel.*—Room without bath, for one person, $2 to $3 a day; for two persons, $4 to $5 a day. Room with bath, for one person, $5 a day; for two persons, $7 a day.

*Mosier Hotel.*—European plan, $1 to $3 per day, with Silver Moon Restaurant attached at very reasonable rates. Located at Ninth and Pine Streets.

*Hotel Rozier.*—Opposite Exposition Building, Olive and Thirteenth Streets. Rooms without bath, $1 to $2 a day.

*Mammoth Hotel Company.*—S. E. Corner Olive and Twelfth Streets. Can accommodate two thousand five hundred guests per day at rates from fifty cents to $1.50 per day.

*The Inside Inn.*—With a capacity for five thousand five hundred people, is within the Exposition Grounds, erected under a contract with the Exposition Management, stipulating its rates.

This Hotel offers five hundred rooms at $1 per day, five hundred at $1.50 a day, five hundred at $2 a day, and the remainder, which are larger, with baths, at higher rates. The Napoleon Bonaparte, the Forest City, the Fraternal, the University, the Kenilworth, the American, the Epworth, the Grand View, the States, the Oakland, the Iowa, the Guaranty, the West Park, the Christian Endeavor, the Visitors, and others, with a capacity of from five hundred to five thousand guests, are within easy walking distance of the World's Fair gates. In fact, we have hotels, boarding-houses, apartment houses, and rooming-houses, all over the city, of respectable character and on the street-car lines.

An impression prevails that there may be lack of accommodation at reasonable prices. Not only will there be sufficient room for all who come, but the rates will be reasonable. We appeal to the profession of the country to give information respecting the accommodations in a spirit of fairness and justice to St. Louis based upon the above facts.

St. Louis is prepared to care for and welcome all comers and to show them the grandest Universal Exposition of the world's resources and products in the history of man.

The Fourth International Dental Congress is now an assured success. Many foreign nations have signified their intention to take part in this Congress. The Islands of the Pacific Ocean will be well represented. Their assembling in the centre of this great republic should stimulate every American dentist to action, and each individual of this great profession should feel under obligations to help to push the Congress to a successful issue. We have our professional record to maintain and act the part of host. As a consequence we should endeavor to sustain the reputation of American hospitality.

Here is the birthplace of the Dental College, the most of the inventors, the mechanical geniuses, and the men who have brought about the wonderful advances in our great profession.

We trust the profession of America will take hold with their accustomed vigor, let nothing be undone that will be for the good of the profession, and carry it out with a most liberal spirit to a surprising conclusion.

A list of hotels, boarding-houses, rooming-houses and private homes, with their rates appended, will be furnished by the Committee to all asking for same. Any other information will be freely given by corresponding to any of the following committee:

D. O. M. LeCron, *Chairman*, Missouri Trust Building.
Max Fendler, *Secretary*, Missouri Trust Building.
H. F. D'Oench.
George H. Gibson.
S. H. Voyles.
Orme H. Manhard.
Joseph G. Pfaff.
G. L. Kitchen.

---

### FOURTH INTERNATIONAL DENTAL CONGRESS, SECTION VI., ORTHODONTIA.

The programme of the Section on Orthodontia of the Fourth International Dental Congress is as follows:

"Irish Types of Malocclusion," W. Booth Pearsall, Dublin.

"Nasal Obstructions and Mouth-Breathing, with Special Reference to Malocclusion of the Teeth," J. Sim Wallace, London.

"A Contribution to the Treatment of Short Bite and Jump Bite Cases," William Slocum Davenport, Paris.

"Nature as a Regulator and our Duty as her Assistants," L. C. Bryan, Basel, Switzerland.

"A Proposal for an International Nomenclature for the Various Forms of Malocclusion," John E. Grevers, Amsterdam, Holland.

"The Study of the Etiology of Anomalies of Human Teeth," Jose J. Rojo, Mexico City.

Essay, M. Chiwaki, Tokyo, Japan.

"The Correction of Deformities in Fractures of the Nose," Francisque Martin, Lyons, France.

"Prognathous Forms and their Orthopædic Treatment," Dr. Scroeder, Greifswald, Germany.

Essay, Franz Zeliska, Vienna.

Essay, Hopewell Smith, London.

"Malocclusion: Class II. and its Divisions," Edward H. Angle, St. Louis.

"Mesial Position of the First Molars in Class I.," Robert Dunn, San Francisco.

"Principles and Methods of Retention in Orthodontia," Calvin S. Case, Chicago.

"Spreading the Maxilla *versus* Spreading the Arch," R. Ottolengui, New York City.

"Art," Edmund Wuerpel, St. Louis.

Essay, Herbert A. Pullen, Buffalo.

Essay, S. Merrill Weeks, Philadelphia.

Essay, G. V. I. Brown, Milwaukee.

"A Method of determining the Normal Arch and its Application in Orthodontia," Charles A. Hawley, Columbus.

EDWARD H. ANGLE, *Chairman.*
MILTON T. WATSON, *Secretary.*

---

### NATIONAL ASSOCIATION OF DENTAL EXAMINERS.

THE National Association of Dental Examiners will hold their annual meeting in the Coliseum Building, corner of Thirteenth and Olive Streets, St. Louis, Mo., on the 25th, 26th, and 27th of August, beginning promptly at 10 A.M. Telephone and telegraph offices in the building. Hotel accommodations will be secured for the members. Special railroad rates will be secured for those in the East desiring to attend, trains leaving on the 23d from New York.

The Committee on Railroad Accommodations for the East have made arrangements for fast through Pullman service to St. Louis from New York with the Delaware and Lackawanna Railroad. Two special Pullman cars will leave New York Tuesday, August 23, at 10 A.M. The cost of our excursion including berth each way will be $35.50. A proportionate reduction is made for those going from Buffalo, Toledo, Fort Wayne, and cities on the line connecting with the Wabash Railroad. To those desiring to go on the special cars, send notice to Charles A. Meeker, D.D.S., Secretary of the National Association Dental Examiners, or to Guy Adams, General Passenger Agent of the Delaware and Lackawana Railroad. Accommodations have been secured for the National Association of Dental Examiners at the Franklin Hotel, northwest corner of Sarah and Westminster Place, with rates from $1.50 to $6.00 per day, European plan. Hotel first-class. Secure rooms by writing to E. C. Dunnavant, St. Louis Service Company, Seventh and Olive Streets, St. Louis, Mo.

CHARLES A. MEEKER, D.D.S.,
*Secretary.*

---

### "F. D. I." FÉDÉRATION DENTAIRE INTERNATIONALE.

THE next (fourth annual) meeting will be held in the Coliseum Building, St. Louis, Mo., August 26 and 27, 1904. The first session will convene under the presidency of Dr. Charles Godon, at 11 A.M. There will be a meeting of the Executive Council on Thursday, the 25th, at the Hotel Jefferson, at 10 A.M.

The Section on Education will meet at 3 P.M. Friday; the Section on Hygiene and Public Dental Service, at 3 P.M. Friday; the Section on International Dental Press, at 4.30 P.M. Friday.

The officers of the Sections are:

*Education.*—President, Dr. T. W. Brophy; Vice-Presidents, Dr. E. C. Kirk, Dr. W. B. Paterson, and Dr. O. Zsigmondy; Secretaries, Dr. M. Roy and Dr. R. B. Weiser.

*Hygiene and Public Dental Service.*—President, Dr. W. D. Miller; Vice-Presidents, Dr. Cunningham, Dr. Forberg, Dr. Jenkins, and Dr. Rose; Secretaries, Dr. R. Heide, Dr. Sauvez, and Dr. R. B. Weiser.

*Commission of the International Dental Press.*—President, Dr. E. Forberg; Dr. A. W. Harlan; Secretary, Dr. E. Papot.

*Executive Council.*—President, Dr. Charles Godon; Vice-Presidents, Dr. A. W. Harlan, Dr. W. D. Miller; Secretary, Dr. E. Sauvez; Treasurer, Dr. F. Aguilar.

*Members.*—Dr. George Cunningham, Dr. E. Forberg, Dr. R. B. Weiser, Dr. J. E. Grevers, Dr. F. Heisse, Dr. Klingelhofer.

On behalf of the Federation,

A. W. Harlan,

*Vice-President.*

1122 Broadway, New York City.

---

### SUPREME CHAPTER, DELTA SIGMA DELTA.

The twentieth annual meeting of the Supreme Chapter, Delta Sigma Delta Fraternity will be held Wednesday, August 31, 1904, at St. Louis, Mo. George E. Hunt, 131 E. Ohio Street, Indianapolis, is chairman of the Committee on Arrangements.

---

### RESOLUTIONS AMERICAN DENTAL TRADE ASSOCIATION.

Whereas, In the estimation of our Association, which is composed of the representative manufacturers of and dealers in dental supplies in the United States, the amendment to patent laws introduced in Senate Bill 4256, House Bill 6771, will remove a class of patents which have long caused annoyance to the dental profession; therefore, be it

"*Resolved,* That the American Dental Trade Association heartily endorse the amendment and urge favorable action thereon, and that the Secretary be instructed to notify the Committees having the amendment in consideration of such action.

"But no patent shall be granted upon any art of treating human disease or disability, or attached to be used in the treatment of human disease, or attached to the human body and used as a substitute for any lost part thereof, or upon any art of making such device unless such device is adopted to be put on the market and sold."

LEE S. SMITH,
*Secretary.*

---

### THE INTERSTATE DENTAL FRATERNITY.

THE Interstate Dental Fraternity will hold its annual meeting at St. Louis on Tuesday, August 30, 1904. The business meeting will be at 3 P.M., to be followed by a banquet.

The committee in charge are Dr. Burton Lee Thorpe, Chairman; Dr. Edward Everett Haverstick, and Dr. Ernest P. Dameron. Members may procure their banquet tickets in advance by remitting to Dr. E. E. Haverstick, 346 N. Boyle Avenue, St. Louis.

R. M. SANGER,
*National Secretary.*

---

### PROGRAMME OF THE F. D. I.

THE International Dental Federation will carry out the following programme, in the Coliseum, St. Louis, on Friday, August 26, at 11 A.M., Dr. Charles Godon presiding.

Address of Welcome, by Dr. William Conrad, of St. Louis.

Response by Dr. Godon.

Address by a representative of the Louisiana Purchase Exposition.

Address by the Mayor of St. Louis.

Response by Dr. H. A. Smith, of Cincinnati, Ohio.

Short addresses by the representatives of the Foreign Countries present; after which the President's Address, by Dr. Charles Godon.

Report by the Secretary-General, Dr. E. Sauvez.

Adjournment, subject to the call of the President.

*Commission on Education.*—Professor Truman W. Brophy, of Chicago, presiding. Will meet in the Coliseum Building, St. Louis, Mo., August 26, at 2 P.M.

Address by the President, Dr. Brophy.

Dr. Charles Godon will give a *résumé* of the status of Dental Education in France.

Dr. Mitchell, of London, will make some observations on technical education.

Dr. Gordon White, of Nashville, Tennessee, will make a short address on the present status of Dental Education in the United States.

Dr. R. B. Weiser, of Vienna, Austria, will give an address on education.

Drs. H. L. Banzhaf, of Milwaukee, C. N. Johnson, of Chicago, M. W. Foster, of Baltimore, and W. E. Boardman, of Boston, will also read papers.

*Commission on Hygiene and Public Dental Service.*—Professor W. D. Miller, of Berlin, presiding. The meeting will convene Friday, August 26, at 2 P.M.

Address by Professor Miller, on Dental Hygiene in Germany.

Dr. George Cunningham, of Cambridge, England, will present a report on Public Dental Service, by the late Dr. J. Franck, of Vienna, Austria, with comments.

Dr. E. Forberg, of Stockholm, will give a paper on Hygiene and Public Service.

Other papers will be presented by members of the Commission.

*Commission on International Dental Press.*—Dr. E. Forberg, of Stockholm, Sweden, presiding. The meeting will convene Friday, August 26, at 4.30 P.M. in the French Building at the Fair Grounds.

Address by Dr. E. Forberg, President.

Paper by Dr. A. W. Harlan, New York, "The advantages of an International Review."

The programme for Saturday will be published and distributed early Saturday morning.

Dr. Conrad has secured the use of the French Building for the members of the F. D. I., from 4 to 6 P.M., Friday, August 26.

The Executive Council will meet at 6.30 P.M. in the Hotel Jefferson.

Members of the dental profession affiliated with the dental societies of the United States, or in foreign countries, are cordially invited to join any or all of the sections of the International Dental Federation.

On behalf of the Federation.

A. W. Harlan,
*Vice-President.*

1122 Broadway, New York City.

